# Toxicity Profile of New Therapies in Metastatic Urothelial Carcinoma and Its Impact on Treatment Selection

**DOI:** 10.3390/cancers17213523

**Published:** 2025-10-31

**Authors:** Patricia Guerrero, Carlos González-Merino, Coral García de Quevedo, José Daniel Subiela, Pilar Sotoca, Juan Carlos Calvo, Carolina Bueno, Adriana García, Inmaculada Orejana, Alberto Artiles, Pablo Gajate

**Affiliations:** 1 Department of Medical Oncology, Ramón y Cajal University Hospital, M-607, Km. 9, 100, Fuencarral-El Pardo, 28034 Madrid, Spain; cgmerino@salud.madrid.org (C.G.-M.); cgquevedosuero@salud.madrid.org (C.G.d.Q.); pilar.sotoca@salud.madrid.org (P.S.); jcalvop@salud.madrid.org (J.C.C.); agarciagoni@salud.madrid.org (A.G.); pablo.gajate@salud.madrid.org (P.G.); 2 Department of Urology, Ramón y Cajal University Hospital, M-607, Km. 9, 100, Fuencarral-El Pardo, 28034 Madrid, Spain; josedaniel.subiela@salud.madrid.org (J.D.S.); albertocristo.artiles@salud.madrid.org (A.A.); 3 Department of Urology, Infanta Sofía Hospital, Paseo Europa, 34, San Sebastián de los Reyes, 28702 Madrid, Spain; carolina.bueno@salud.madrid.org; 4 Department of Medical Oncology, MD Anderson Cancer Center, Calle Arturo Soria, 270, 28033 Madrid, Spain; i.orejana@mdanderson.es

**Keywords:** new therapies, urothelial carcinoma, toxicity profile, therapeutic sequencing

## Abstract

**Simple Summary:**

The introduction of new therapies has revolutionized the landscape of urothelial carcinoma, with a significant impact on patient survival. However, the optimal therapeutic sequence has yet to be defined and may be influenced by the toxicity profile of available treatments. The aim of this review is to synthesize the current evidence on the toxicity associated with each drug, describe management strategies, and analyze how these factors may affect the selection of future therapeutic lines.

**Abstract:**

Metastatic urothelial carcinoma (mUC) is a common tumor associated with high mortality. To date, the standard chemotherapy-based treatment has yielded suboptimal outcomes, characterized by limited survival and a substantial impact on patients’ quality of life. The introduction of new therapies has significantly improved overall survival (OS) and progression-free survival (PFS) rates. However, bringing novel treatments into clinical practice comes with an unfamiliar toxicity profile that may influence the choice of systemic therapy. The aim of this review is to analyze the toxicity of these new therapies and reflect on the role of appropriate management in treatment selection and planning of therapeutic sequencing in patients with mUC.

## 1. Introduction

Metastatic urothelial carcinoma (mUC)is among the ten most frequent solid tumors, with higher incidence rates in Southern European countries, including Spain, where it ranks fifth [1]. For decades, the standard first-line treatment has been platinum-based chemotherapy [2], with very limited prognosis: OS rates are approximately 14 months for cisplatin-treated patients and 10 months for those treated with carboplatin, with 5-year survival rates of only 5–10% [3,4].

The introduction of immunotherapy (ICI), targeted therapy, and antibody-drug conjugates (ADCs) has marked a paradigm shift in the mUC treatment landscape, with significant improvements in OS and PFS. Currently, there are three first-line systemic treatment options, all with level IA evidence: enfortumab-vedotin plus pembrolizumab, cisplatino plus gemctiabina followed by avelumab maintenance and cisplatino plus gemcitabine plus nivolumab [5]. However, these new therapies have been associated with non-negligible toxicity-related discontinuation rates (around 10–15%) [6,7,8,9,10], reaching up to 20–35% when used in combination [11,12]. The purpose of this review is to examine the adverse events associated with new therapies and discuss the importance of their clinical management in optimizing systemic treatment for patients with mUC.

### Review Design and Literature Search

This article is a narrative review based on a comprehensive analysis of the most relevant literature concerning the management of advanced urothelial carcinoma and treatment-related toxicities. A non-systematic search of the PubMed/MEDLINE and Embase databases was conducted, focusing on publications from 2020 to 2025. Studies were selected based on their clinical relevance and contribution to understanding toxicity profiles and therapeutic decision-making. The toxicity was graded based on the version of CTCAE in effect at the time each pivotal study was conducted.

## 2. New Therapies

### 2.1. Antibody–Drug Conjugates (ADCs)

#### 2.1.1. Enfortumab-Vedotin

Enfortumab-vedotin (EV) is an ADC composed of a fully human monoclonal antibody targeting nectin-4 and a microtubule-disrupting chemotherapeutic agent, monomethyl auristatin E (MMAE) [13,14]. The phase III EV-301 trial randomized patients who progressed on first-line platinum-based chemotherapy followed by maintenance ICI to receive EV versus chemotherapy (taxanes or vinflunine). Both PFS and OS were superior in the ADC arm: 5.55 months vs. 3.71 months (HR 0.62; *p* < 0.001) and 12.88 months vs. 8.97 months (HR 0.70; *p* = 0.001) [6].

A total of 301 patients were treated with EV. Among these, 34% (102/301) required dose reductions due to toxicity, and 17% (51/301) permanently discontinued the drug for this reason [6]. To understand the toxicity profile of EV-treated patients, it is crucial to highlight the drug’s mechanism of action. Nectin-4 is an adhesion protein overexpressed not only in tumor cells of the urothelial tract but also in healthy tissues (especially skin, salivary and mammary glands, and gastrointestinal tract) [14]. After binding to nectin-4, EV is internalized into the cytoplasm and releases MMAE, which disrupts the cell cycle by interacting with microtubules. This induces apoptosis, and the free MMAE may affect neighboring cells, extending its cytotoxic effect [15,16].

The most frequent toxicity was dermatologic, with 43.9% (132/301) experiencing a maculopapular rash. Severe cutaneous adverse events such as bullous epidermolysis or erythema multiforme occurred in 20.3% (61/301) of patients, and grade 3–4 skin reactions were observed in 14.5% (44/301) [6]. Alopecia and pruritus were reported in 47% (141/301) and 34.5% (104/301) of patients, respectively [6]. Dermatological toxicity is primarily due to the expression of Nectin-4 in normal skin cells, leading to off-target effects of the ADC [17].

Peripheral neuropathy, another frequent and dose-limiting toxicity, occurred in 46.3% (139/301), with 3.7% (11/301) experiencing grade ≥ 3 events [6]. Neuropathy was the main reason for dose reductions (7.1%, 21/301), discontinuation (15.5%, 47/301), or treatment withdrawal (2.4%, 7/301) [6]. Most cases were predominantly sensory and affect de lower limbs [18]. Since axonal transport depends on microtubules disrupted by MMAE this explains the underlying pathophysiology [19].

Hyperglycemia occurred in 6–7% (18–21/301), more frequently among patients with BMI ≥ 30 kg/m^2^. Most events were grade 3 hyperglycemia (57.8% of these cases, 10–12/18–21), and one resulted in fatal diabetic ketoacidosis [6,20]. The mechanism of EV-induced hyperglycemia is not fully clarified [21].

Finally, EV was associated with hematologic toxicity, primarily anemia (19.9%, 60/301) and grade 3 neutropenia (6.1%, 18/301) [6], likely due to MMAE’s effect on rapidly dividing hematopoietic cells—similar to its impact on keratinocytes.

#### 2.1.2. Trop-2 Inhibitors

Sacituzumab govitecan (SG) is an ADC targeting Trophoblast Cell Surface Antigen 2 (Trop-2). According to results from the phase III TROPiCS-04 trial, SG showed a higher objective response rate (ORR) compared to standard chemotherapy in pretreated patients (23% vs. 14%), although no statistically significant differences were observed in terms of PFS and OS [7].

Trop-2 is a transmembrane glycoprotein expressed in most epithelial cells, and its signaling promotes cellular proliferation and migration [22]. It is overexpressed in many solid tumors, including urothelial tumors [23,24,25], and this has been associated with poorer prognosis [26,27,28]. Trop-2 is linked to the active metabolite of irinotecan (SN-38), which is released inside the cell after ADC internalization and exerts a cytotoxic effect [29,30].

SG toxicity is mainly hematologic and gastrointestinal, as these are tissues composed of rapidly dividing cells and therefore more susceptible to SN-38’s inhibition of topoisomerase I [31].

More than half of SG-treated patients experienced grade ≥ 3 adverse events, affecting up to 67% (239/357) of patients, with neutropenia being the most common (35%, 125/357). Toxic deaths were reported in 7% (25/357) of SG-treated patients, compared to 2% (7/354) in the control arm; among these deaths, 64% (16/25) were attributed to infection in the setting of neutropenia [7]. Other grade ≥ 3 toxicities included anemia (8%, 29/357) and gastrointestinal effects such as vomiting and diarrhea (5–8%, 18–29/357) [7].

Other Trop-2–targeting ADCs, such as datopotamab deruxtecan and sacituzumab tirumotecan, have demonstrated different toxicity profiles compared to SG in early-phase trials [32,33].

#### 2.1.3. HER2 Inhibitors

Numerous studies have explored the use of anti-HER2 therapy in the setting of mUC with HER2-positive expression. While tyrosine kinase inhibitors (TKIs) with anti-HER2 activity have not shown clinical benefit [34,35,36,37], ADCs targeting HER2 have demonstrated somewhat more promising results in pretreated patients.

In the phase II DESTINY-PanTumor02 trial, the efficacy of trastuzumab deruxtecan (T-DXd) was evaluated based on HER2 expression levels. The greatest benefit was observed in patients with HER2 IHC 3+, where the overall response rate (ORR) reached 61.3% (95% CI: 49.4–72.4), median PFS was 11.9 months (95% CI: 8.2–13.0), and OS was 21.1 months (95% CI: 15.3–29.6). In patients with HER2 IHC 2+, the ORR was lower (26.5%) [38].

Disitamab vedotin (DV) has been evaluated in a phase II clinical trial in patients with mUC and HER2 overexpression, defined as IHC 2+ or 3+. It was associated with an ORR of 51.2% (95% CI: 35.5–66.7%), a PFS of 6.9 months (95% CI: 5.6–8.9), and an OS of 13.9 months (95% CI: 9.1–not estimable) [39]. In patients without HER2 overexpression (IHC 0 or 1+), the reported ORR was lower (26.3%) [40]. Further clinical trials are needed to provide sufficient evidence to support its incorporation into routine clinical practice.

HER2 is a transmembrane glycoprotein belonging to the epidermal growth factor receptor (EGFR) family, with tyrosine kinase activity that promotes angiogenesis, differentiation, and cell proliferation [41]. Its overexpression has been primarily associated with gastric and breast cancers [42], where HER2 has become a well-established therapeutic target [43,44,45]. HER2 overexpression has also been observed in a notable proportion of urothelial tumors (approximately 10–20%) [46,47].

For T-DXd, the most frequent toxicities were gastrointestinal and hematologic. Nausea occurred in 51.2% (19/36) of patients and diarrhea in 31.7% (11/36). Hematologic toxicities included anemia in 29.3% (10/36) and neutropenia in 26.8% (10/36). However, the most serious adverse event was interstitial lung disease (ILD), which led to treatment discontinuation in 9.8% (4/36) of cases and was the most common cause of treatment withdrawal, as well as the only reported treatment-related death in the mUC cohort. This toxicity is not due to Her2 expression in lung tissue, but rather stems from the tissue damage caused by the activation of alveolar macrophages upon uptake of the ADC. The typical onset occurs between 4 and 6 months after the start of treatment [38].

For patients treated with DV, the discontinuation rate was higher, reaching 25.6% (11/43), mainly due to peripheral neuropathy manifested as hypoesthesia. This was also the most frequently reported toxicity, occurring in 60.5% of patients (26/43; 23.3% (10/43) grade ≥ 3). The mechanism underlying the peripheral neuropathy induced by this drug is the same as in the case of enfortumab vedotin, as it causes disruption of the axonal microtubules. It tends to appear 3–4 months after starting treatment. Other relevant toxicities included hematologic events such as leukopenia (55.8%, 24/43) and neutropenia (41.9%, 18/43), although these were mostly not clinically significant [39].

### 2.2. Checkpoint Inhibitors (ICI)

ICI treatment has become established as a therapeutic option in mUC. Both pembrolizumab and atezolizumab are recommended as second-line treatments for patients who have progressed on platinum-based chemotherapy regimens [2,48,49,50]. Although neither has shown superiority over chemotherapy in the first-line setting, both are considered valid treatment options for frail patients or those ineligible for platinum, due to their favorable safety profile [51,52]. Later, the JAVELIN Bladder 100 study demonstrated a significant OS benefit with avelumab as maintenance therapy in patients who achieved disease control after platinum-based chemotherapy, with a median OS of 21 months versus 14 months in the control group [9,53].

To understand the toxicity profile of ICIs, it is essential to delve into their mechanism of action. Immune checkpoint proteins (PD-1 on T lymphocytes; PD-L1 on other cells, overexpressed in tumor cells) physiologically inhibit the body’s immune response. Inhibiting these proteins activates T cells capable of attacking tumor cells. For this reason, drugs targeting PD-1, PD-L1, and CTLA-4 have been developed to stimulate the body’s immune response against tumors.

This mechanism may result in immune-mediated toxicities involving inflammation in any organ. Although rare, some of these toxicities can be severe. In the phase III trial comparing pembrolizumab versus chemotherapy, pneumonitis was the only treatment-related adverse event that resulted in death [48]. These adverse effects have been reported less frequently with atezolizumab [49]. Other, milder immune-related toxicities, such as skin, thyroid, pituitary (hypophysitis) or hepaticdisorders, may also occur with any ICI treatment.

### 2.3. Targeted Therapy

#### FGFR Inhibitors

In pretreated patients with mutations in the fibroblast growth factor receptor (FGFR), the use of FGFR inhibitors (FGFRi) has shown promising results. In the phase III THOR trial, erdafitinib was compared to chemotherapy in patients with mUC and FGFR mutations who had received one or two prior lines of therapy, including ICIs. Results favored erdafitinib, with a median OS of 12.1 months vs. 7.8 months (HR 0.64; *p* = 0.005) and a median PFS of 5.6 vs. 2.7 months (HR 0.58; *p* < 0.001) [10].

FGFR signaling promotes cellular growth and migration. Erdafitinib acts as a pan-FGFR inhibitor, blocking this pathway and thereby halting cell proliferation.

Erdafitinib is associated with one of the highest toxicity rates among mUC treatments, with a discontinuation rate of 14.1% (19/136). The THOR study reported adverse reactions of any grade in 98.5% of patients (134/136), more than in the chemotherapy arm (97.3%: 127/130). Nearly half of the patients experienced grade ≥ 3 adverse events (45.9%, 62/136), the most common being palmar-plantar erythrodysesthesia (9.6%, 13/136), which manifests as redness and pain in the extremities and significantly affects quality of life [10].

Other notable grade ≥ 3 events included skin disorders (11.9%, 16/136), nail disorders (11.1%, 15/136), and stomatitis (8.1%, 11/136), all attributable to the rapid turnover of epithelial cells. Ocular toxicity (4.4%, 6/136), including central serous retinopathy (CRS) and dry eye, was also significant. This toxicity is explained by the presence of FGFR receptors in the retina, retinal pigment epithelium, and cornea. When these receptors are inhibited by the drug, the normal pumping mechanism is disrupted, leading to the accumulation of subretinal fluid. It tends to appear within the first few weeks after starting treatment [10].

Other frequent toxicities associated with erdafitinib included diarrhea (62%, 84/136) and hyperphosphatemia (80%, 109/136), which, although characteristic, rarely caused symptoms or reached clinically significant levels [10].

### 2.4. Combination Therapies Involving ICI

Until now, we have discussed the results of ICI monotherapy, either as first-line treatment for frail patients or in the post-platinum setting. However, an increasing number of studies are evaluating ICIs in combination with other therapies, sometimes increasing the toxicity profile previously described.

Recent data on the effectiveness of EV combined with ICI suggest a potential shift in the first-line treatment standard for mUC. The phase III EV-302 clinical trial showed promising results in terms of PFS, OS, and ORR, favoring EV + pembrolizumab over platinum-based chemotherapy: PFS (12.5 vs. 6.3 months; HR 0.45; *p* < 0.001), OS (31.5 vs. 16.1 months; HR 0.47; *p* < 0.001), ORR (67.7% vs. 44.4%; *p* < 0.001) [11].

This combination substantially increases the risk of peripheral neuropathy, affecting up to half of the patients, of whom 3.6% (16/442) experienced grade ≥ 3 neuropathy. It was the most frequent cause of treatment discontinuation (10.7%; 47/442). However, severe skin toxicity did not increase with combination therapy: grade ≥ 3 skin events were reported in 7.7% (34/442) of patients treated with EV + ICI [11] compared to 14.5% (41/301) for EV monotherapy [6]. Additionally, the combination did not increase the incidence of severe hyperglycemia [54]. The treatment discontinuation rate in the experimental arm was 35.0% (155/442), and the dose reduction rate was 40.7% (180/442).

For patients who are not candidates for EV + pembrolizumab (e.g., those with poorly controlled diabetes mellitus), the combination of ICI with platinum-based chemotherapy is a valid alternative. Results from the CheckMate 901 study demonstrated OS and PFS benefits in the arm combining nivolumab with cisplatin + gemcitabine versus cisplatin + gemcitabine alone: OS 21.7 vs. 18.9 months (HR 0.78; *p* = 0.02); PFS 7.9 vs. 7.6 months (HR 0.72; *p* = 0.001). The most relevant toxicity in the chemo + ICI arm was hematologic [12].

Other combinations of ADCs with ICIs have also been explored. T-DxT + nivolumab yielded a median OS of 11.0 months (95% CI: 7.2–NE). The most significant toxicity in this case was pneumonitis [55]. DV + toripalimab showed an ORR of 76% in the overall population and 83.3% in HER2 IHC 2+/3+ patients, with a PFS of 9 months (95% CI: 5.8–12.1). The toxicity profile was manageable, with no reported adverse events requiring dose reduction [56,57]. A phase III clinical trial is currently recruiting to evaluate DV + pembrolizumab versus chemotherapy in previously untreated patients, although no results have been published yet [58].

Cohort 3 of the TROPHY-U-01 trial evaluated SG combined with pembrolizumab in platinum-refractory patients. An ORR of 41% (95% CI: 26.3–57.9), median PFS of 5.3 months (95% CI: 3.4–10.2), and median OS of 12.7 months (95% CI: 10.7–NE) were observed. The most relevant grade 3 toxicity was again hematologic [59]. The most relevant toxicity of each drug is summarized in Table 1.

## 3. Discussion

The emergence of new drugs in the mUC scenario has significantly expanded the available treatment options. Faced with this broader therapeutic range, the question arises whether we should prioritize initial efficacy in all cases, even at the cost of limiting access to subsequent lines, or whether the choice of first-line treatment should be adapted to the clinical profile of each patient.

Although the phase II clinical trial TROPHY-U-01 showed favorable data in terms of response and survival in favor of SG in patients with mUC [60], recent data from the phase III study TROPiCS-04 comparing SG versus chemotherapy in pretreated mUC patients did not demonstrate a benefit for SG. Nevertheless, SG activity was evidenced through a higher ORR, and the authors conclude that early complications related to drug toxicity could have influenced the efficacy results [7]. On the other hand, in patients with metastatic breast cancer (MBC) treated with SG, a much lower incidence of severe adverse effects and toxic deaths has been observed. In the phase III TROPiCS-02 study, conducted in patients with MBC and hormone receptor-positive status, febrile neutropenia was reported in only 5% of cases (14/272), discontinuations due to grade ≥ 3 toxicity were 6% (16/272), and a single toxic death due to neutropenic colitis was recorded (1/272) [61]. In contrast, in the TROPiCS-04 study, carried out in pretreated mUC patients, febrile neutropenia reached 12% (43/357), treatment interruptions due to adverse events were 11% (39/357), and 15 toxic deaths (15/357) were reported, most associated with infections in the context of neutropenia [7]. These differences highlight the impact of the patient’s clinical profile on treatment tolerance. While in the TROPiCS-02 study, most patients were younger (74.2% were under 65 years old), in TROPiCS-04, the average age was higher (66 years), and the general condition more compromised: up to 75% had at least one poor prognosis factor according to Bellmunt criteria (anemia, ECOG ≥ 1, or liver involvement) [7]. This contrast underscores the need to adapt therapeutic strategies not only to the drug but also to the patient’s clinical context, a key aspect in trial design and in defining the maximum tolerated dose.

These differences become even more evident in routine clinical practice. Real-world studies in mUC more faithfully reflect the fragility of the population we treat. The SAUL study (a single-arm trial evaluating atezolizumab safety in pretreated patients) recruited 1004 patients, including populations usually excluded from pivotal trials. It was observed that up to 10% had ECOG 2, 5% had renal insufficiency with creatinine clearance <30 mL/min, 7% had received two or more prior systemic treatment lines, and up to 14 patients had brain metastases [62]. Similarly, real-world studies of patients treated with EV show a population with similar characteristics: 19.3% with ECOG ≥ 2, 59% with at least two prior treatment lines, 24% with three or more, and 80% with visceral metastases (31% hepatic and 49% non-hepatic) [63].

Beyond the obvious differences observed between patients with different solid tumors, it is worth exploring whether certain subgroups of mUC patients could benefit from personalized therapeutic alternatives based on their clinical profile. Until now, the Galsky and Gupta criteria [64,65] have guided the selection of patient candidates for cisplatin and carboplatin in first-line treatment, respectively. However, with the incorporation of new therapeutic alternatives, these criteria are insufficient for adequate selection, especially in the first-line context where multiple treatment strategies exist with comparable levels of evidence [5].

Currently, we have at least three valid first-line strategies for mUC: while the combination of EV + pembrolizumab yields the best data in terms of OS, PFS, and ORR [11] and is the preferred option in most clinical guidelines [5,66,67], chemotherapy followed by maintenance avelumab allows for maintaining long-term clinical benefit in approximately 20% of patients without cumulative toxicity, which has a great impact on quality of life [53,68]. On the other hand, the combination of cisplatin, gemcitabine, and nivolumab also shows promising efficacy results with the possibility of benefiting from ICI from the start, without depending on an initial response as in the case of avelumab [12].

Currently, there is no validated tool to determine the most appropriate treatment for each patient. However, some groups have proposed approaches that can help identify the most suitable option for each case. One example is the EVITA criteria (acronym for EV-Ineligible criTeriA), designed to define those patients in whom the combination of EV + pembrolizumab might not be an optimal option. They include patients with at least two of the following characteristics: glycated hemoglobin levels ≥ 8% (or fasting glucose ≥ 150 mg/dL on two consecutive occasions), creatinine clearance less than 45 mL/min, motor or sensory neuropathy ≥ grade 2, ECOG ≥ 2, or corneal or retinal abnormalities [69]. Additionally, multiple subgroup analyses based on clinical characteristics have been carried out with the objective of identifying patients who might obtain greater benefit from certain treatments (for example, those with disease limited to lymph nodes, visceral metastases, or who are eligible for cisplatin). However, none of these analyses have been conclusive [11,12,53].

Traditionally, efforts have been made to find molecular alterations that can predict response to chemotherapy. Somatic alterations in genes involved in DNA damage repair (DDR) have been associated with better outcomes in patients treated with platinum, suggesting their potential utility as biomarkers to select patient candidates for chemotherapy [70,71]. Attempts have also been made to subclassify urothelial tumors according to molecular subtypes, since it has been observed that basal tumors are associated with a higher response to chemotherapy than luminal tumors [72,73,74,75]. From this line of research emerges the molecular consensus classification of bladder cancer, which defines 6 molecular subtypes that could be used in the future to validate predictive biomarkers of response [76]. The expression of genes such as RAD51, IFNγ, and CHEK1 has also been associated with better outcomes in patients with mUC treated with chemotherapy [77]. However, none of these markers has yet reached sufficient validation to be applied in clinical practice. Their integration together with clinical parameters and the tumor’s taxonomic subtype could increase their predictive value for response to chemotherapy [77].

Efforts have also been made to define predictive biomarkers of response to treatment with ICIs. Alterations in DDR have been independently associated with greater efficacy of anti-PD-1/PD-L1 treatment in patients with mUC, with an even more robust benefit observed in those with known or likely pathogenic mutations in DDR [78]. Additionally, a correlation has been described between PD-L1 expression and the clinical benefit of ICIs in mUC. A meta-analysis demonstrated a higher ORR and a significant reduction in the risk of death and progression in patients with PD-L1-positive compared to those with PD-L1-negative [79]. However, significant responses have been observed in both groups, and studies present considerable methodological heterogeneity by using different cut-off points to define PD-L1 positivity, which limits the clinical applicability of this marker. On the other hand, tumor mutational burden (TMB) has been significantly associated with greater benefit from ICIs in terms of ORR, PFS, and OS; a benefit that is even greater if associated with PD-L1 expression [80,81,82,83]. Therefore, the combination of both biomarkers could increase their predictive value.

In the context of patients treated with EV, nectin-4 amplification appears consistently associated with a higher response rate to treatment: in the study carried out by Klümper et al., 96% of patients carrying nectin-4 amplification showed objective response, compared to 32% in the group without amplification (*p* < 0.001). Additionally, nectin-4 amplification correlated with a significant reduction in risk of death (HR 0.08; 95% CI: 0.02–0.34), independently of other clinical prognostic factors [84]. Other studies have supported this hypothesis [85], suggesting the potential value of nectin-4 expression as a predictive biomarker of response to treatment with EV in patients with mUC.

However, we still do not have sufficient scientific evidence to establish solid predictive factors of response, nor well-defined clinical profiles that guide the choice of the most appropriate treatment. In the absence of such information, the optimization of therapeutic outcomes must be based on the understanding and effective management of the toxicity profile associated with each therapeutic regimen. Although the arrival of new therapies to clinical practice has meant a great clinical benefit, it is accompanied by a toxicity profile that may be unknown to us and that we must learn to manage. It is essential to implement a multidisciplinary management approach for these patients from the initiation of new therapies, with the aim of anticipating the onset of severe toxicities and managing them appropriately should they occur. This approach not only optimizes current treatment but can also condition the viability of future therapeutic lines. In the data published in the EV-302 clinical trial, treatment-related adverse events (TRAEs) caused treatment interruption in up to 35% of cases [11]. Learning to manage toxicity can prevent us from withdrawing a drug that might be effective. Sometimes, there are treatments aimed at the different TRAEs. In the absence of available therapeutic measures, clinical suspicion and early diagnosis of them can help us take measures, such as dose reduction or therapeutic rest. The management of TRAEs associated with new therapies is summarized in Table 2, Table 3, Table 4, Table 5, Table 6, Table 7, Table 8 and Table 9.

This prospective study evaluated nerve conduction in patients with mUC treated with EV. It was observed that early alterations in nerve conduction, after the first month of treatment, were associated with the later development of clinical peripheral neuropathy. Additionally, patients who developed neuropathy had better ORR (83.3% vs. 40%), greater disease control (100% vs. 65%), and longer PFS [18]. Similar findings have been described in patients with skin toxicity, which suggests that the efficacy of EV could be related to the amount of MMAE released and, therefore, to nectin-4 expression, also present in the skin [86,87].

Currently, there are no effective treatments to prevent or reverse EV-induced neuropathy. The ASCO guidelines discourage any drug as prophylaxis [88] and only duloxetine has shown some clinical benefit in symptomatic cases [89,90,91]. The most effective strategies aim to reduce the dose or interrupt treatment. Therefore, an early electrophysiological study can identify patients at risk of developing neuropathy and guide dose adjustments to avoid severe toxicity that leads to drug withdrawal. Recently published data indicate that dose reduction of EV does not compromise treatment efficacy [92]. In this way, the effect of EV could be maximized in the long term, since, as mentioned previously, a higher response rate is achieved in patients who develop this type of toxicity. The method for reducing the dose of each drug is summarized in Table 10.

As we have mentioned previously, platinum-based chemotherapy continues to be a valid treatment regimen in patients who have not received it previously or in those in whom more than one year has passed between the end of treatment and progression (rechallenge) [2,5]. The neurological toxicity induced by EV is high and is potentiated when combined with pembrolizumab. By becoming a first-line standard of treatment based on the EV-302 study [11], we could be reclassifying patients who were initially eligible for cisplatin as non-eligible according to Galsky criteria [64]. Moreover, in patients who have already progressed after a first line of treatment, we will face worse ECOG and greater fragility, making them less candidates to be treated with any type of chemotherapy. These circumstances can negatively affect the efficacy of subsequent treatment lines, highlighting the importance of proper management of treatment-related adverse effects to optimize clinical outcomes. There are likely intermediate cases in which cisplatin could be maintained with dose reduction or closer monitoring [93,94].

The debate arises as to whether we will have better clinical outcomes in patients treated with EV+ pembrolizumab followed by platinum-based chemotherapy or if we should change the order of regimens. Although the EV-302 clinical trial has shown clear superiority of EV in combination with pembrolizumab compared to platinum-based chemotherapy treatment, it is unknown what impact the subsequent application of this treatment in the first line may have. Recently published real-world data show favorable results in patients treated with platinum-based chemotherapy after having previously received EV+ pembrolizumab: ORR of 50%, PFS of 4.4 months (95% CI, 3.7–7.8 months) and OS of 12 months (95% CI, 9.7–17 months). Although the duration of the response was modest, the results suggest that platinum-based chemotherapy retains relevant antitumor activity even after EV+ pembrolizumab [95]. On the other hand, the AVENANCE study shows real-world data of the reverse sequence: among patients treated with platinum-based chemotherapy and maintenance avelumab, second-line treatment with EV was associated with an OS of 41.5 months, superior to the subgroup that received another chemotherapy as second line (24.5 months) [96]. These data suggest that the integration of EV after maintenance avelumab can optimize results in real practice. However, caution is warranted when interpreting evidence from real-world data. Patients included in these studies often have more heterogeneous characteristics than those in controlled clinical trials, including a broader range of prior therapies, varying ECOG performance status, and higher burdens of visceral disease or comorbidities. Real-world outcomes can also be influenced by missing data, censoring, and potential survival bias. Therefore, while these studies provide valuable insights into treatment effectiveness and safety in routine practice, their results should be interpreted carefully within the context of these inherent limitations.

Sufficient evidence has not yet been generated to answer this question. More studies and real-world data are needed to help us understand the impact of new treatments and the management of their toxicity when determining the therapeutic sequence to follow in patients with mUC.

## 4. Conclusions

The arrival of new therapies to clinical practice opens a hopeful horizon for patients with mUC. Learning about the toxicity profile of each drug and its proper management can help us optimize the treatment of each patient and determine the therapeutic sequence to follow to achieve greater survival benefit.

## Figures and Tables

**Table 1:** Safety summary.

	Incidence	Most Relevant	Leading to Dose Reduction	Leading to Treatment Withdrawal	Leading to Death
Enfortumab- Vedotin (*n* = 301) [6]	70.9%	Dermatological (14.2%) Peripheral neuropathy (5.9%) Hyperglycemia (3.7%) Ocular disorders (0.7%) Hematological (14%) Decreased neutrophil count (6.1%)	34.1%	17.2%	3.7%
Sacituzumab-Govitecan (*n* = 357) [7]	67%	Neutropenia (35%) Anemia (13%) Diarrhea (15%) Nausea and vomiting (6%)	37%	11%	7%
Pembrolizumab (*n* = 370) [51]	16%	Hepatitis (1%) Colitis (1%) Pneumonitis (1%) Arthritis (1%) Rash < 1%	None	11%	<1%
Atezolizumab (*n* = 119) [52]	16%	Colitis (1%) Rash (1%) Hepatitis (1%)	None	8%	<1%
Avelumab (*n* = 350) [9,53]	16.6%	Colitis (0.6%) Arthritis (0.6%) Hypothyroidism (0.3%) Rash (0.3%)	None	11.9%	<1%
Erdafitinib (*n* = 136) [10]	45.9%	Hyperfosfatemia (5.2%) Stomatitis (8.1%) Palmar-plantar erythrodysesthesia (9.6%) Nail disorder (11.1%) Skin disorder (11.9%) Eye disorder (4.4%)	65.9%	8.1%	4.4%
Disitamab- Vedotin (*n* = 43) [39]	58.9%	Peripheral neuropathy (23.3%) Neutropenia (14%)	Not reported	25.6%	None
Trastuzumab-Deruxtecan (*n* = 36) [38]	41.5%	Neutropenia (10.9%) Anemia (10.9%) Pneumonitis (0.4%)	36.6%	9.8%	2.4%

**Table 2:** Dermatological toxicity.

Grade	Definition	Management	Symptomatic Treatment
G1	<10% body surface area Asymptomatic or mild symptoms	Continue therapy	Emollients, topical steroids, oral antihistamines
G2	10–30% body surface area Moderated symptoms (impacting daily activity)	Continue therapy *	Emollients, topical steroids, oral antihistamines
G3	>30% body surface area Self-care limiting symptoms	Hold until ≤G1 Reintroduce at dose level −1	Refer to dermatology
G4	Severe, potentially life-threatening	Discontinue permanently	Hospital admission

* Grade 2 with progressive worsening or fever should be managed as Grade 3. Data on toxicity management, dose modifications, and treatment interruptions are based primarily on the official drug label. Toxicities were graded according to the version of CTCAE in effect at the time each label was developed. Clinical guidance for monitoring and supportive care is also aligned with ESMO^2^ recommendations where applicable).

**Table 3:** Neuropathy.

	Definition	Management	Symptomatic Treatment
G1	Asymptomatic or mild symptom. Clinical or diagnostic observations only	Continue therapy	Duloxetine has shown some clinical benefit in symptomatic cases 90–92
G2	Moderate symptoms limiting instrumental activities of daily living	Hold until ≤G1 Reintroduce at dose level −1	
G3	Severe symptoms limiting self-care activities	Discontinue permanently	
G4	Severe, potentially life-threatening	Discontinue permanently	

**Table 4:** Pneumonitis.

	Definition	Management			Symptomatic Treatment		
G1	Asymptomatic Radiologic findings only	EV Continue therapy	T-DXd Hold until complete resolution <28 days: same dose >28 days: level −1	ICI Delay treatment Monitor symptons every 2–3 days	EV None	T-DXd Corticosteroids (0.5–1 mg/kg/day)	ICI None
G2	Symptomatic, not limiting self-care (cough, mild/moderate dyspnea)	EV Hold until ≤G1 Reintroduce at dose level −1	T-DXd Discontinue permanently	ICI Hold until ≤G1	Corticosteroids (1 mg/kg/day)		
G3	Severe, limiting self-care (oxygen requirement)	Discontinue permanently			Hospitalize Corticosteroids (1–2 mg/kg/day) Cover with empirics antibiotics (if ICI are identified as the underlying cause)		
G4	Severe, life-threatening (mechanical ventilation)						

**Table 5:** Ocular toxicity.

	Definition	Management Baseline Ophthalmologic Evaluation Is Mandatory, Followed by Monthly Assessments for the First 4 Months and Every 3 Months Thereafter
G1	Asymptomatic or mild symptom. Clinical or diagnostic observations only, or abnormal Amsler grid test.	Ophthalmologic exam (OE) in <7 days: - If no signs of ocular toxicity are found on OE, continue therapy - If OE diagnosis shows keratitis or retinal alteration (including CSR), temporarily hold until resolved. Reintroduce at same dose or level −1
G2	Moderate symptoms, limitation of age-appropriate activities of daily living	Hold and refer for OE immediately: - If no ocular toxicity are found, reintroduce at same dose or level −1 after symptoms resolution - If ocular toxicity are found (keratitis or CSR) but it resolve within less than 4 weeks, reintroduce at level −1 after symptoms resolution
G3	Clinically significant symptoms, limitation of self-care activities of daily living without threat to vision	Hold and refer for OE immediately: If resolved within 4 weeks, reintroduce at level −2 after symptoms resolution
G4	Vision-threatening consequences, blindness (20/200 or worse)	Discontinue permanently

**Table 6:** Hyperphosphatemia.

	Definition	Management	Symptomatic treatment
G1	Serum phosphate: 5.6–6.9 mg/dL	Continue therapy	Phosphate binder until ≤G1
G2	Serum phosphate: 7.0–8.9 mg/dL	Continue therapy or dose level −1 if: - Serum phosphate ≥ 7.0 mg/dL is maintained for >2 months - Electrolyte disorders related to prolonged hyperphosphatemia occur.	
G3	Serum phosphate: 9.0–10.0 mg/dL	Hold until ≤G1 Reintroduce at same dose or level −1 if: - Serum phosphate ≥ 9.0 mg/dL is maintained for >1 months - Electrolyte disorders related to prolonged hyperphosphatemia occur.	
G4	Serum phosphate: 7.0–9.0 mg/dL	Hold until ≤G1 Reintroduce at level −1 if or discontinue permanently if: - Serum phosphate ≥ 10.0 mg/dL is maintained for >2 weeks - Electrolyte disorders related to prolonged hyperphosphatemia occur.	

**Table 7:** Neutropenia.

	Definition	Management	
		Sacituzumab-Govitecan	Anti-Trop2
G1	1500 ≤ LLN/mm^3^	Continue therapy	Continue therapy
G2	1000–1500/mm^3^	Continue therapy	Continue therapy
G3	500–1000/mm^3^	Grade 4 lasting ≥ 7 days Grade 3 and fever ≥ 38.5 °C Grade 3–4 causing a delay of 2 to 3 weeks until recovery to ≤Grade 1: - First occurrence: Granulocyte colony-stimulating factor (G-CSF) - Second occurrence: Reduce dose by 25% - Third occurrence: Reduce dose by 50% - Fourth occurrence Discontinue treatment	Hold until ≤G2. Reintroduce: - If T < 38 °C: at same dose - If T > 38 °C: level −1
G4	<500/mm^3^		Hold until ≤G2. Reintroduce at level −1

**Table 8:** Gastrointestinal toxicity.

	Definition		Management
	Vomiting	Diarrhea	
G1	Intervention not indicated	<4 stools per day	Continue therapy
G2	Outpatient intravenous hydratation	4–6 stools per day Limiting instrumental activities of daily living	Continue therapy
G3	Hospitalization indicated: tube feeding or total parenteral nutrition	≥7 stools per day Limiting self-care activities of daily living	Any Grade 3–4 vomiting or diarrhea that is not controlled with antiemetics and antidiarrheals (persists for more than 48 h despite optimal management) At the time of scheduled treatment causes a delay of 2 to 3 weeks until recovery to ≤Grade 1 - First occurrence: Reduce dose by 25% - Second occurrence: Reduce dose by 50% - Third occurrence: Discontinue treatment - Fourth occurrence: Discontinue treatment
G4	Life-threatening consequences	Life-threatening consequences	

**Table 9:** Immune-mediated toxicity.

	Definition	Management	Symptomatic Treatment
G1	Asymptomatic or mild symptoms Clinical or diagnostic observations only Intervention not indicated	Continue therapy with close monitoring *	None
G2	Moderated symptoms Limiting instrumental activities of daily living	Hold ICI and resume when symptoms and/or laboratory values revert until ≤G1	Corticosteroids (0.5–1 mg/kg/day)
G3	Limiting self-care activities of daily living	Hold until ≤G1 rechallenging may be offered (caution is advised)	Corticosteroids (1–2 mg/kg/day) They should be tapered over the course of at least 4–6 weeks If symptoms do not improve with 48–72 h, infliximab may be offered for some toxicities.
G4	Severe symptoms Potentially life-threatening	Discontinue permanently	

* Except for: Neurologic: Myasthenia gravis, Guillain–Barré síndrome, transverse mielitis, encephalitis, neuropathies. Hematologic: Immune thrombocytopenic purpura (ITP), autoimmune hemolytic anemia (AIHA), hemolytic uremic syndrome (HUS), severe thrombocytopenia. Cardiac: Myocarditis, arrhythmias, heart failure, elevated cardiac biomarkers, electrocardiogram abnormalities.

**Table 10:** Toxicity-based dose reductions.

	Initial Dose	Dose Reductions
Enfortumab-Vedotin	1.25 mg/kg/day	1.00 mg/kg/day –> 0.75 mg/kg/day –> 0.5 mg/kg/day
Sacituzumab-Govitecan	10 mg/kg/day	7.5 mg/kg/day –> 5 mg/kg/day
ICI	Pembrolizumab: 200 mg/day Atezolizumab: 1200 mg/day Avelumab: 800 mg/day	Dose adjustments are not recommended
Erdafitinib	8 mg/day Increase the dose to 9 mg after 14–21 days if no toxicity is observed.	8 mg/day –> 6 mg/day –> 5 mg/day –> 5 mg/day
Trastuzumab- Deruxtecan	5.4 mg/kg/day	4.4 mg/kg/day –> 3.2 mg/kg/day
